# The Impact of Nursing Characteristics and the Work Environment on Perceptions of Communication

**DOI:** 10.1155/2012/401905

**Published:** 2012-02-22

**Authors:** Dana Tschannen, Eunjoo Lee

**Affiliations:** ^1^Division of Nursing Business and Health Systems, School of Nursing, University of Michigan, 400 N Ingalls, Room 4152, Ann Arbor, MI 48109-5482, USA; ^2^College of Nursing, Kyungpook National University, Daegu 702-701, Republic of Korea

## Abstract

Failure to communicate openly and accurately to members of the healthcare team can result in medical error. The purpose of this study was to explore the impact of nursing characteristics and environmental values on communication in the acute care setting. Nurses (*n* = 135) on four medical-surgical units in two hospitals completed a survey asking nurses' perceptions of communication, work environment, and nursing demographics. LPNs perceived significantly higher levels of open communication with nurses than did RNs (*P* = .042). RNs noted higher levels of accuracy of communication among nurses than did LPNs (*P* < .001). Higher experience levels resulted in greater perceptions of open communication. Only environmental values (e.g., trust, respect) were a significant predictor of both openness and accuracy of communication. These findings suggest understanding the environment (e.g., presence or absence of trust, respect, status equity, and time availability) is a foundational step that must occur before implementing any strategies aimed at improving communication.

## 1. Introduction

A significant cause of medical error in health care is poor communication [1, 2]. For the past three years, miscommunication has been identified as one of the most frequently identified root causes of sentinel events reported to The Joint Commission, with 82% of the sentinel events in 2010 identifying communication as the primary root cause [3]. According to Rucker and colleagues, up to 75% of clinical decisions are made without all pertinent clinical information [4]. Differences in status and discipline may be part of the confounding factors associated with poor communication. This includes various job categories (supervisor/supervisee), expertise level (novice/expert), and discipline (doctor/nurse) [2]. 

Although variations in status and discipline are abundant in the healthcare environment, it is critical for all members of the healthcare team to communicate effectively with one another, despite these differences. In an effort to understand how status and discipline differences may impact perceptions of communication, the purpose of this study was to explore the impact of nursing characteristics (e.g., job category, education, experience, and expertise) on perceptions of communication in the acute care setting, while also considering the impact of the work environment.

## 2. Literature Review

The act of communication between nurses and physicians is a central activity in healthcare, and a failure to communicate has been linked with poor quality and patient errors [5]. Effective communication and collaboration among nurses and physicians has been shown to result in improved quality of care [6, 7], increased patient and professional satisfaction [6, 8], and greater intent to stay [8, 9]. Specifically, the presence of poor communication among nurses and physicians may result in an almost doubled risk for mortality and length of stay among intensive care unit (ICU) patients [8, 10, 11]. Manojlovich and colleagues, while surveying nurses in 25 ICUs, found timeliness of communication to be inversely correlated with pressure ulcer development (*r* = −.38, *P* = .06) [12]. In addition, higher variability of understanding—which can occur with a variety of education and experience levels—was significantly correlated with ventilator associated pneumonia (*r* = .43, *P* = .03). 

Current research evaluating the impact of nursing characteristics on communication has resulted in mixed findings. Miller and colleagues, while examining the presence of individual characteristics and perceptions of nurse-physician interactions, found nurses with greater than six years of experience rated openness of communication and problem solving higher than less experienced nurses (*P* = .04) [13]. Foley and colleagues found a significant relationship between nurse-physician relationships and nursing expertise and the number of professional certifications (*P* = .05) [14]. In contrast, Mark and colleagues evaluated the relationship between nurse staffing, professional practice, and several patient outcomes and found no significant relationship between nurse staffing variables (education, experience, and skill mix) and professional practice [15].

Although nursing characteristics such as education and expertise level may determine levels of communication and collaboration (i.e., physicians may respect nurses who are more educated), values supported in the environment in which care is delivered may also impact communication patterns. When the values of the organization include trust, respect, and teamwork, collaborative relationships are more likely to ensue. According to Schmalenberg and colleagues, who conducted interviews with physicians and nurses, environmental values play a role in fostering the development of effective communication/collaboration [16]. One interviewee described collaboration as “a prevailing unit and organizational norm based on mutual trust, respect, teamwork, and open communication.” Findings from focus groups of nurses and physicians conducted by Simpson and colleagues identified an agreement among participants that many interactions and experiences with one another over time were the basis for trust and confidence in one another [17].

In summary, communication among the healthcare team is critical for optimal patient outcome. The current literature has failed to identify specific communication strategies that have consistently impacted quality of care and patient safety [18]. This may be due to the failure to consider individual characteristics, such as education, experience, and expertise levels, as well as the values present in the environment. In addition, little work has been done beyond the critical care areas—ICUs, emergency rooms, and operating rooms. For this reason, the purpose of this study was to identify the relationship between individual nursing characteristics (education, experience, expertise, and job type), environmental values, and perceptions of communication with the healthcare team in the acute care setting.

## 3. Methods

### 3.1. Design

This study used a cross-sectional, descriptive design with a convenience sample of four in-patient medical surgical units in two Midwestern hospitals. All nurses employed on the units providing direct patient care were asked to participate. Nurses who did not perform direct patient care were excluded. A total of 161 registered nurses (RNs) and 18 licensed practice nurses (LPNs) were eligible for study participation. Based on a power analysis (multiple regression with 11 predictors) with an *α* of 0.05, medium effect size (*f* ^2^ = .15), and power (1 − *β*) = .80, 123 respondents were needed for the analysis. The number of questionnaires returned was 135, with response rates for the units ranging from 69% to 82% (overall response rate of 76%). Approval for the study was obtained from the institutional review board for each institution.

### 3.2. Data Variables and Survey Instrument

The survey tool used to identify perceptions of communication was a modified version of Shortell's Organizational Management in the Intensive Care Unit Survey [19]. The entire survey included 44 questions asking nurses' perceptions of communication, collaboration, the environmental values present in their respective units, as well as nursing demographic information. For the purpose of this study, the questions regarding communication, environmental values, and nursing demographics were used.

#### 3.2.1. Communication

Communication was measured by two dimensions: openness and accuracy. Communication openness refers to “the degree to which physicians or nurses are able to ‘say what they mean' when speaking with members of the other group, without fear of repercussions or misunderstanding” [19, page 712].” Four questions on the survey instrument addressed the openness of communication among nurses and four additional questions considered the openness of communication between nurses and physicians. Each item was measured by a 7-point Likert scale with anchors 1 (strongly disagree) to 7 (strongly agree). Communication accuracy refers to the “degree to which nurses and physician believe in the accuracy of the information conveyed to them by the other party [19].” Four questions on the survey instrument addressed the accuracy of communication among nurses and four additional questions considered accuracy of communication between nurses and physicians. Each item was measured by a 7-point Likert scale (1, strongly disagree, to 7, strongly agree). Validity and reliability of the instrument had been previously reported [19]. Reliability estimations in this study also supported consistency in the items: open communication among nurses (*α* = 0.89) and between nurses and physicians (*α* = .92), accuracy of communication among nurses (*α* = .79) and between nurses and physicians (*α* = .84). 

#### 3.2.2. Nursing Characteristics

Nursing characteristics included in this study were education, years of experience, and self-reports of expertise. Level of education was measured categorically with the following options being present: diploma, associate's degree, bachelor's degree master's degree, or higher. Nursing experience was measured through a single-item question: How many years have you been working in your current job category? The final measure of nursing expertise required the nurses to identify their perceived level of expertise on a 10-point scale with anchors novice (1) to expert (10). Respondents were asked to circle the number on the scale that best reflects his/her level of expertise. Other nursing characteristics included in the study were job category (e.g., LPN or RN), unit of employment, and shift worked (e.g., day, evening, night, or rotating).

#### 3.2.3. Environmental Values

The previous literature has identified environmental values as important precursors to the development of effective communication and collaborative relationships, including trust, respect, power equity, and time availability. Questions related to each of these values was developed and measured by a single question on a 7-point Likert scale with anchors strongly agree (1) to strongly disagree (7). Data from this study supported a highly positive correlation between the four factors, as noted by the following correlation values: trust and respect (*r* = .82), trust and time (*r* = .54), trust and status (*r* = .67), respect and time (*r* = .60), respect and status (*r* = .66), and finally time and status (*r* = .62) (*P* = .001 for each bivariate association). This supported the development of an overall environmental value variable, which was the combined average of each of the unit value items (per nurse). Reliability estimation for the environmental value variable was considered well above the acceptable range (*α* = 0.88).

### 3.3. Procedure

Prior to distribution of the survey, nurses were presented with a 10-minute overview of the study. This overview was given to each unit at four different times of the day, in an effort to attain maximum participation. Upon completion of the in-service, each nurse received a copy of the survey. A reminder was placed in each nurse's mailbox two weeks after the initial survey distribution in an effort to increase response rate. A secure box was also placed in the nursing lounge of each unit for completed surveys.

### 3.4. Analysis

Data were analyzed with SPSS 18.0. Descriptive statistics were used to examine the demographics of nurses; analysis of variance (ANOVA) and chi-square tests were performed to test homogeneity of unit characteristics. To identify the difference in communication between nursing characteristics, *t*-tests were performed. Hierarchical multiple regression analysis was conducted to identify predictors of openness and accuracy of communication. A test for multicollinearity was conducted using tolerance and VIF; no multicollinearity was identified. Residual analysis identified a normal distribution, linearity of residual, and homoscedasticity of errors. A significant value below 0.05 was considered statistically significant.

## 4. Findings

Nursing respondents (*n* = 135) were split nearly equally between Hospital A (*n* = 74, 55%) and Hospital B (*n* = 61, 45%). The majority of the nurses were RNs (*n* = 119, 88%) while 15 were LPNs (11%). Seventy-three (54.1%) nurses had earned an associate/diploma degree and 58 (43%) had a baccalaureate degree. Sixty-eight nurses worked the day shift and 43 nurses worked the night shift. Work experience as nurses was on average 12.30 years, ranging from 6 weeks to 46 years. Self-rating of expertise level was 6.98, with a range of 1 (novice) to 10 (expert).

Comparisons of nurse educational level, work experiences, and expertise levels by study units revealed no difference in educational level and expertise level (Table 1). A significant difference in work experiences was noted, with Unit A having the highest work experiences as nurses, followed by Unit B (*P* = .046).

### 4.1. Differences in the Perception of Communication

As noted in Table 2, nurses (e.g. RNs and LPNs) perceived communication to be more open among nurses than between nurses and physicians (*t* = 10.227,  *P* < .001). However, nurses perceived that communication was more accurate with physicians than with nurses (*t* = 2.18, *P* = .031).

When comparing openness and accuracy of communication between job category (e.g., RN and LPN) (Table 2), LPNs perceived significantly higher levels of open communication with nurses than did RNs (*P* = .042). In contrast, RNs noted higher levels of accuracy of communication among nurses than did their LPN counterparts (*P* < .001). No significant difference between LPNs and RNs was noted in openness and accuracy of communication with physicians.

### 4.2. Predictors of Openness of Communication

Hierarchical multiple regression analysis was conducted to identify the variables which predicted openness of communication (Table 3). Individual nursing characteristics were entered in Step 1, explaining 8.4% of the variation in open communication among nurses (nonsignificant). After entry of the unit and environmental value variables (Step 2), the total variance explained by the model was 33.1% (*F* = 5.25, *P* < .001). Only environmental values (*P* < .001) and unit (*P* = .009 and *P* = .011) were significant predictors of open communication among nurses. Specifically, the more positive the environmental values (e.g., high trust, respect, etc.), the greater the perception of communication openness among nurses. In addition, unit was a significant predictor, such that nurses on Units C and D noted lower levels of communication openness than the referent group (Unit A).

A second analysis, with dependent variable open communication between nurses and physicians, was computed with independent variables job category, education, shift, experience, and expertise entered in Step 1. Only education level was predictive of open communication between nurses and physicians. Specifically, higher education levels were associated with greater perceptions of communication openness with physician colleagues. In Step 2, the environmental values and unit variables were entered (Table 3). The final model explained 64.6% of the variance (*F* = 19.396, *P* < .001). The significant predictors of open communication with physician included the evening shift (*P* = .048), years of experience as a nurse (*P* = .035), environmental values (*P* < .001), and unit (unit 4, *P* = .022). Education levels were no longer a significant predictor. Nurses working the evening shift perceived lower openness of communication compared to day shift nurses. Nurses with more years of experience noted higher levels of openness in communication (*B* = .125, *P* = .035). In addition, a more positive environment was predictive of greater openness in communication (*B* = .797, *P* < .001). 

### 4.3. Predictors of Accuracy of Communication

Hierarchical multiple regression analysis was also used to identify the variables which predicted accuracy of communication (Table 4). Individual nursing characteristics entered in Step 1  explained 23% of the variance in accuracy of communication among nurses (*F* = 5.03, *P* < .001). An additional 11.1% of variation was explained with the inclusion of the unit and the environmental value variables entered in Step  2. The final model (*F* = 5.345, *P* < .001) explained 34.2% of the variance in accuracy of communication (among nurses). Job category (e.g., LPN) (*P* = .001), shift (*P* < .001) (e.g., night), and environmental values (*P* < .001) were significant predictors. Specifically, LPNs perceived less accuracy in communication among the nursing staff than their RN counterparts. Nurses working night shift identified greater accuracy in communication than their day shift colleagues. A more positive environment was associated with less accuracy in communication among nurses.

Another analysis, with dependent variable accuracy of communication between nurses and physicians, was computed with the following independent variables: job category, education, shift, expertise, years of experience, environmental values, and unit. The first model (Step 1) included the nursing characteristics variables and explained 14.1% of the variance in the dependent variable (*F* = 2.80, *P* = .01). The unit and environmental values variables, entered in Step 2, explained an additional 19.3%, for a total of 33.4% of variance explained (*F* = 5.235, *P* < .001). Significant predictors included shift (night,  *P* < .001, and rotating, *P* = .028) and environmental values (*P* < .001). Specifically, nursing staff working night and rotating shifts identified greater accuracy in communication between nurses and physicians than did day shift nurses. In addition, a work environment with greater trust, respect, time, and status equity was predictive of lower accuracy of communication between nurses and physicians (*P* < .001).

## 5. Discussion

This study sought to identify the relationship between individual nursing characteristics and perceptions of communication with the healthcare team. Findings revealed a significant relationship between some of these variables. Overall, nurses (both RNs and LPNs) reported greater openness among nurses than between nurses and physicians. In contrast, they reported communication between nurses and physicians to be more accurate than among the nursing team.

Significant variation in perceptions of openness and accuracy of communication were identified between RNs and LPNs. RNs identified significantly more accurate communication among nurses whereas LPNs identified significantly greater communication openness. This may in part be related to the role expectations of the LPN and RN. The LPN, due to licensure restrictions, must be assigned to an RN, and therefore frequent interaction among the nurse dyad (RN-LPN) is required. Due to an increase in interactions with RNs, LPNs may note greater inaccuracies in communication among the team. RNs—in contrast to LPNs—can work autonomously due to his/her greater scope of practice, and therefore, do not rely on communication from others to determine patient care needs.

Experience level was also predictive of communication. Specifically, years of experience of the nurse was significantly related to openness of communication among nurses and physicians. This may be in part due to the need for frequent interaction for antecedents of effective communication, including trust and respect, to develop [17]. Also, nurses with greater years of experience may be viewed as having greater expertise among physician colleagues, especially in an acute care environment where physician colleagues may rotate monthly. Higher levels of education were associated with greater perceptions of nurse-physician communication, but when the environment was considered, this was nonsignificant. This sheds some light on the importance of the context of the environment.

Unit of employment was predictive of openness and accuracy of communication. This may be related to the fact that Unit C and D had the lowest average for years of experience and self-reported expertise, which was shown to predict openness in communication between nurses and physicians. Nurse shift was also significantly associated with perceptions of communication openness and accuracy. Specifically, night shift nurses identified greater levels of accuracy of communication among nurses, and between nurses and physicians compared to nurses on the day shift; in contrast, evening shift nurses identified lower levels of communication openness than the day shift. These findings may be related to the presence of physicians on these shifts. At night, less staff (both nursing and medical) are present, which may result in a greater need to work together to ensure optimal care delivery; communication must be more accurate for timely implementation of appropriate interventions.

The values present in the environment were predictive of all four outcome variables (e.g., openness/accuracy of communication among and between nurses and physicians). As expected, when positive values, such as trust, respect, and status equity, are present on the environment, openness in communication among the healthcare team ensues. This finding is similar to other studies [16, 17] which have noted the impact of these variables on effective communication. Work environments that foster trust, respect, status equity, and time availability create an atmosphere were communication can flourish. Interestingly, the same values that fostered open communication seem to reduce accuracy in communication. According to the study findings, greater presence of the work environment values was associated with less accuracy in communication. One potential reason for this may be that staff working in a positive environment (e.g., trust and respect present) is more willing to state their opinions about patient care needs; Jones and George found trust among team members fostered greater willingness to share information freely among the team [20]. In contrast team members who do not feel valued or believe information may be used inappropriately are less willing to share pertinent information [21]. In such an environment (e.g., low trust and respect), staff may be less likely to share their thoughts, and instead state only facts that are fully supported.

There are two noteworthy limitations of this study. The data for this study came from four acute care units located in one of two Midwestern Hospitals, thus generalizability is limited to similar medical-surgical acute care units. In addition, the survey captured *perceptions* as opposed to *actual* communication patterns. Therefore, the actual accuracy and openness of communication was not measured. To study actual communication patterns would involve an extensive observational study and would be very complex and costly.

## 6. Conclusions

Communication among the healthcare team is critical for optimal patient care. When communication is not open and accurate, medical errors result. Findings from this study identified nursing characteristics (e.g., experience, unit, shift worked) and the environmental context as essential for open communication. Understanding the environment (e.g., presence or absence of trust, respect, status equity, and time availability) is a foundational step that must occur before implementing any strategies aimed at improving communication. A failure to understand the environment may in part explain why no one strategy has been shown to consistently improve nurse-physician communication [18]. Further research is needed to determine the best strategies for developing trust and respect among the healthcare team. For example, the development of trust requires consistent interaction. Current work environments where staff—both nursing and physicians—rotate, create less opportunity for interaction.

One potential strategy for increasing interactions among the healthcare team would be through consistent team nursing (e.g., nurses on a team work the same shifts/days). This would result in frequent interactions where the antecedents to effective communication (e.g., trust/respect) could develop among the nursing team, and subsequently with other members of the healthcare team.

Another possible strategy for improving communication among the healthcare team includes multidisciplinary education. According to a position paper on interdisciplinary education and practice from the American Association of Colleges of Nursing (AACN), programs and curricula must be developed that incorporate opportunities for collaborative learning and decision making [22]. Educating nurses and physicians together may result in greater role clarity, shared decision making, and more positive attitudes towards collaboration.

Additional strategies for improving communication include encouraging open dialogue, collaborative rounds, and engagement on interdisciplinary committees [23]. This can provide opportunities for discussing problem areas and collaboratively determining strategies to reduce miscommunication. Regardless of strategies implemented, all healthcare professionals have a common commitment to serving the patient and assisting them in reaching their optimal level of functioning. This can result when communication among the healthcare team is open and accurate.

## Figures and Tables

**Table 1:** Homogeneity test by unit characteristics (*n* = 135).

	Unit A M (SD)	Unit B M (SD)	Unit C M (SD)	Unit D M (SD)	*F*/*χ*²	*P*
Experience as nurses (years)	15.86	12.81	8.37	11.24	2.741	.046
Expertise level	7.28	7.02	6.79	6.76	.490	.690
Educational level	N (%)	N (%)	N (%)	N (%)		
Diploma/Associate	23 (57.5)	16 (48.5)	18 (56.3)	16(55.2)	.671	.880
BSN and over	17(42.5)	17(51.5)	14 (43.8)	13 (44.8)		

**Table:** (a)

	Within nursing Mean (SD)	Between DR and RN Mean (SD)	*t*	*P*
Open communication	5.74 (1.00)	4.45 (1.30)	10.227	.000
Accuracy of communication	2.68 (.80)	2.82 (.91)	2.18	.031

**Table:** (b)

	RN (*n* = 119) M (SD)	LPN (*n* = 135) M (SD)	*t*	*P*
Open communication with nurses	5.75 (1.01)	6.32 (0.7)	−2.051	.042
Open communication with physicians	4.44 (1.30)	4.60 (1.48)	−.432	.667
Accuracy of communication with nurses	3.29 (0.59)	2.56 (0.4)	4.542	.000
Accuracy of communication with physicians	3.28 (0.65)	3.04 (0.51)	1.350	.179

**Table 3:** Hierarchical multiple regression analysis predicting openness of communication (*n* = 135).

	Open communication among nurses				Open communication between nurses and physicians			
	*β*	*t*(*P*)	*β*	*t*(*P*)	*β*	*t*(*P*)	*β*	*t*(*P*)

Constant	5.542	.000	4.983	.000	3.007	.000	0.361	.375
LPN(RN = 0)	0.637	.042	0.325	.245	0.141	.724	−0.47	.072
Education	0.14	.419	0.018	.908	0.454	.049	0.189	.197
Nights(day = 0)	0.021	.914	−0.098	.572	0.033	.897	−0.119	.475
Evenings	−0.49	.087	−0.473	.061	−0.315	.400	−0.477	.048
Rotating	−0.6	.137	−0.213	.553	0.052	.922	−0.036	.916
Expertise	0.006	.917	0.001	.982	0.08	.302	−0.029	.569
Experience	0.009	.891	−0.035	.567	0.071	.425	0.125	.035
Environment			0.274	.000			0.797	.000
Unit B (Unit A = 0)			0.13	.537			0.088	.659
Unit C			−0.594	.009			0.397	.064
Unit D			−0.578	.011			0.493	.022
*F(P)*		1.580 (.148)		5.225 (.000)		1.481 (.180)		19.396 (.000)
*R* ^2^		.084		.331		.079		.646
∆*R* ^2^		.084		.247		.079		.567

**Table 4:** Hierarchical multiple regression analysis predicting accuracy of communication (*n* = 135).

	Accuracy communication among nurses				Accuracy communication between nurses and physicians			
	*β*	*t(P)*	*β*	*t(P)*	*β*	*t(P)*	*β*	*t(P)*

constant	2.385	.000	2.952	.000	2.798	.000	3.34	.000
LPN (RN = 0)	−0.994	.000	−0.801	.001	−0.217	.416	0.007	.977
Education	−0.059	.663	0.024	.855	−0.055	.721	0.058	.678
Nights (Day = 0)	0.493	.002	0.549	.000	0.562	.001	0.647	.000
Evenings	0.295	.184	0.311	.142	0.408	.104	0.4	.080
Rotating	0.693	.027	0.577	.057	1	.005	0.724	.028
Expertise	0.03	.520	0.044	.319	−0.025	.629	−0.016	.744
Experience	−0.02	.705	−0.008	.880	−0.009	.883	0.024	.675
Environment			−0.202	.000			−0.241	.000
Unit B (unit A = 0)			−0.098	.583			−0.116	.544
Unit C			0.121	.517			0.375	.065
Unit D			0.1	.600			0.399	.055
*F*(*P*)		5.026 (.000)		5.345 (.000)		2.790 (.010)		5.235 (.000)
*R* ^2^		.231		.342		.141		.334
Δ*R* ^2^		.231		.111		.141		.193
